# Structural Elucidation of a Carnosine-Acrolein Adduct and its Quantification in Human Urine Samples

**DOI:** 10.1038/srep19348

**Published:** 2016-01-19

**Authors:** Vanderson S. Bispo, Ivan P. de Arruda Campos, Paolo Di Mascio, Marisa H. G. Medeiros

**Affiliations:** 1 Departamento de Bioquímica, Instituto de Química, Universidade de São Paulo, São Paulo, São Paulo, Brazil; 2 Instituto de Ciências Exatas e Tecnologia, Universidade Paulista, São Paulo, São Paulo, Brazil

## Abstract

Aldehydes accumulate in inflammation, during myocardial infarction and have been associated with pain symptoms. One pathway of aldehyde detoxification is the conjugation with carnosine. A 3-methylpyridinium carnosine adduct from the reaction of carnosine and acrolein was characterized using extensive spectroscopic measurements. The adduct with urinary concentrations of 1.82 ± 0.68 nmol/mg of creatinine is one of the most abundant acrolein metabolites in urine and opens promising therapeutic strategies for carnosine.

Lipid peroxidation produces a large number of reactive aldehydes as secondary products, including 4-hydroxy-*trans*-2-nonenal (HNE), 2-propenal (acrolein, ACR), and malondialdehyde (MDA)^1^. Many of these aldehydes are cytotoxic and have been reported to form adducts with biomolecules, including glutathione, amino acids, proteins and DNA^2^. Reactive aldehydes can react with DNA^3^ and covalently modify phospholipids, thiols and amine residues to form Michael adducts or can modify lysine residues in proteins through Schiff base formation^4^. Some of these lesions are considered possible pathways for oxidative stress-related diseases such as inflammation, neurodegenerative disorders, cardiovascular diseases, diabetes and cancer^2^,^5^.

A well-recognized pathway of reactive aldehyde detoxification in living cells is the conjugation of these aldehydes with glutathione (GSH) to give Michael adducts. Aldehydes are also detoxified by reduction or oxidation reactions catalyzed by alcohol dehydrogenase, aldo–keto reductase and aldehyde dehydrogenase^6^. In addition, endogenous histidine-containing dipeptides such as carnosine (β-alanyl-L-histidine, CAR), homocarnosine (γ-amino-butyryl-histidine) and anserine (*β*-alanyl-L-1-methylhistidine) have been recognized as detoxifying agents against reactive carbonyl species^7^. Carnosine is found in high concentrations in skeletal muscle as well as in the central nervous system^5^. However, its physiological function is still not fully understood. Though it is widely accepted that carnosine plays an important role as an intracellular buffer^8^, carnosine has also been described as having physiological roles as an anticonvulsant^9^, a quencher of metals^10^ and a promoter of anti-senescence^11^. Carnosine also has the ability to react with α, β-unsaturated aldehydes, including HNE and ACR^5^. In addition, as reviewed by Vistoli and co-workers^12^, carnosine conjugation has been suggested as a mechanism involved in the prevention, inhibition and reversal of the formation of advanced glycoxidation (AGEs) and lipid-oxidation end products (ALEs). In fact, the treatment of obese Zucker rats with carnosine for 24 weeks significantly reduced the incidence of obese-related diseases and prevented the development of dyslipidemia, hypertension and renal injury^13^. Michael adducts formed from the reaction of carnosine with HNE and their corresponding metabolites were identified in rat urine, suggesting that the detoxifying reaction of carnosine occurs *in vivo*^14^ and that carnosine by-products can be used as possible biomarkers of lipid-derived carbonyl stress. Conjugates of carnosine and aldehydes are excreted in urine as carnosine-propanols^6^. Acrolein also forms covalent conjugates with other related histidyl dipeptides, indicating that biological histidyl dipeptides may exert a general detoxifying function by reacting with unsaturated aldehydes^6^. These studies are in agreement with other works that suggest that carnosine, in addition to acting as an intracellular buffer, behaves similarly to GSH in that it may function as a cytosolic scavenger^5^.

Herein, we report the complete characterization of a new product arising from the reaction of carnosine and acrolein. In addition, the biological formation of the CAR-ACR adduct was demonstrated by the detection and quantification of this product in human urinary samples through the development and validation of a new, sensitive method using on-line reverse-phase HPLC coupled with an electrospray ion-trap tandem mass spectrometer (ESI^+^ HPLC-MS/MS). This system allowed for the simultaneous quantitative determination of 3-methylpyridinium carnosine (CAR-ACR), L-carnosine-4-hydroxy-2-hexenal (CAR-HHE) and L-carnosine-4-hydroxy-*trans*-2-nonenal (CAR-HNE) adducts in human urinary samples from non-smoking adults. The work was approved by the Human Ethics Committee of the Institute of Biomedical Sciences of the University of São Paulo. Methods were carried out in accordance with the approved guidelines (Supplementary Methods). Written informed consent was obtained from every urine donor.

The synthesis of the CAR-ACR adduct was performed as described by Carini *et al.*^15^, with modifications. L-Carnosine (45 μmol) was incubated with acrolein (15 μmol) in 1 mL of phosphate buffer, 10 mM, pH 7.0. The solution was mixed at 1000 rpm at 37 °C for 24 h. The adducts were analyzed using an HPLC LC 20 series system (Shimadzu, Kyoto, Japan) coupled to an Amazon Speed electrospray ion-trap mass spectrometer (Bruker Daltonics, Bremen, Germany). A Luna NH_2_ analytical column (150 mm × 3.0 mm i.d., 5 μm, 100 Å, Phenomenex, Torrance, CA, USA) at 25 °C and a flow rate of 0.6 mL/min was used for the analyses. The purifications were performed with a Luna NH_2_ semi-preparative column (250 mm × 10 mm i.d., 100 Å, 10 μm of particle size Phenomenex, Torrance, CA. USA) at 25 °C and a flow rate of 5.5 mL/min. The HPLC mobile phase consisted of 5 mM ammonium formate in water (A) and acetonitrile (B). The analysis and purification of the adduct was performed using a linear elution gradient: 95 to 20% B from 0–25 min, 20 to 5% B from 25–50 min, and 5 to 95% B from 50–60 min to re-equilibrate the column. The fraction eluting at 10 min with *m/z* = 303 was collected, lyophilized and kept at −20 °C (Supplementary Fig. 1). The product was solubilized in D_2_O, DMSO-*d*_*6*_ or pyridine-*d*_*5*_ and characterized by ^1^H 1D, ^1^H-{^1^H} COSY, and ^1^H-{^13^C} HSQC NMR experiments as well as mass spectral analysis (Supplementary Figs 2 and 3). ^1^H NMR δ (DMSO-d_6_): 1.9 (s, 3H, H22), 2.9 (J = 6.5 Hz, t, 2H, H3), 4.7 (J = 6.6 Hz, t, 2H, H2), 6.3 (bs, 1H, H13 or H15), 6.5 (bs, 1H, H13 or H15), 6.8 (bs, 1H, H6), 7.1 (J = 6.2 Hz, t, 1H, H20), 7.4 (J = 7.9 Hz, d, 1H, H19), 7.45 (s, 1H, H14), 7.5 (s, 1H, H16), 8.85 (J = 6.1 Hz, d, 1H, H21), 8.95 (s, 1H, H17), 12.0 (s, 1H, H10); ^13^C NMR δ (DMSO-d_6_): 24.1 (C22), 35.0 (C3), 57.7 (C2), 126.8 (C20), 133.3 (C16), 136.0 (C14), 138.7 (C19), 144.9 (C17), 153.5 (C21). The synthesis and purification of the CAR-HHE and CAR-HNE adducts are described in the SI. The isotope-labeled CAR-HHE-*d*_*5*_ and CAR-HNE-*d*_*11*_adducts were used as internal standards (Supplementary Methods).

Direct infusion-Ion-Trap MS and ESI-Ion-Trap MS_n_ were used for the analysis of the products arising from the reaction of carnosine with HHE and HNE (Supplementary Figs 2 and 3). The reactions gave rise to the same main products described before^16^,^17^,^18^. The molar extinction coefficient of the products were determined from UV measurements as ε_260_ = 2.5037 × 10^2 ^M^−1^cm^−1^ for CAR-HHE and ε_266_ = 1.9212 × 10^2^ M^−1^.cm^−1^ for CAR-HNE (Supplementary Fig. 4). The MS_n_ and NMR analysis of the product with *m/z* = 303 from the reaction used to synthesize CAR-ACR suggested a different structure from that reported in the literature, thus prompting a careful investigation. Previous studies have shown that in aqueous solution, carnosine initially reacts with unsaturated aldehydes to form a Schiff base, which is followed by a Michael addition, cyclization reaction, and the formation of a hemiacetal product^16^. Alternatively, a pyrrole ring^14^ or a picoline ring^18^ may be formed. By applying these mechanisms to the reaction of carnosine with ACR and matching the proposed products with the results of the HPLC-MS/MS (Fig. 1) and ^1^H and ^13^C NMR (Supplementary Figs 5 and 6) experiments, it became clear that the product yielded in our experiments was the one containing a β-picoline moiety. Its exact mass was determined as *m/z* = 303.1415 (Fig. 1). This result is compatible with the molecular formula C_15_H_19_N_4_O_3_ (Compass Isotope Pattern, Bruker Daltonics, Bremen, Germany). The NMR results showed three hydrogens bound to carbons. Each hydrogen was coupled to the next hydrogen at 8.9, 7.1 and 7.4 ppm, respectively, with apparent *J* values *ca.* 6–8 Hz. A further lone hydrogen indicated by an apparent singlet at 9.0 ppm and a methyl group indicated by an apparent singlet at 2.0 ppm, when taken together, suggested a β-picolinium moiety. Furthermore, two hydrogens that were not bound to carbon were indicated by peaks at 6.3 and 6.5 ppm, suggesting that two of the three nitrogen atoms of the molecule were bound to hydrogens (proven by the disappearance of these signals when the compound was diluted in D_2_O–data not shown).

These results led us to conclude that the structure of the product of the reaction between carnosine and acrolein with *m/z* = 303 contained a β-picolinium ring (Fig. 2) corresponding to structure III (Supplementary Fig. 7). A further, more thorough analysis of all of the NMR spectral data supports well this conclusion. Additionally, the UV spectrum of the purified product showed an absorbance at 260 nm, which is also consistent with a picoline chromophore (ε_266_ = 5.18796 × 10^2^ M^−1^cm^−1^–Supplementary Fig. 4C). Thus, our data indicated that similar to the reaction of carnosine with HNE^15^,^17^, the reaction of carnosine with ACR first involves the binding of the distal amino group of carnosine with ACR to form a Schiff base, which then undergoes dehydration and cyclization *via* Michael addition into an imidazole ring. This results in the formation of the unstable intermediate compound at *m/z* = 265, which can react with another acrolein molecule. A cyclization reaction results in the formation of the final product (1-(3-{[1-carboxy-2-(1*H*-imidazol-4-yl)ethyl]amino}-3-oxopropyl)-N-3-methylpyridinium at *m/z* = 303 (Fig. 2). This compound is similar to the one described by Furahata *et al.*^19^, which resulted from the reaction between lysine and acrolein and is suggested to be a potent carcinogen. This product is also similar to various other products formed in the reaction of carnosine with unsaturated aldehydes^6^, though here, we observed a different structure from the one reported before^15^,^16^.

A sensitive methodology based on ESI^+^ HPLC-MS/MS (Supplementary Fig. 8) was developed for the accurate quantification of CAR-ACR, CAR-HHE and CAR-HNE adducts in human urinary samples from non-smoking adults. Supplementary Fig. 9 shows a representative chromatogram of the analysis. The method allowed for the sensitive quantification of 10 pmol of CAR-HHE and 1 pmol of CAR-ACR and CAR-HNE (signal/noise > 6) in urine, with 98% accuracy and variation coefficients (VC) of 10% (Supplementary Fig. 10).

In the urine samples from men, concentrations of 1.82 ± 0.68, 2.30 ± 1.45, 1.28 ± 0.46 nmol/mg of creatinine were found for CAR-ACR, CAR-HHE and CAR-HNE, respectively (Fig. 3). In a recent work, Baba *et al.*^6^ showed that CAR-aldehyde adducts are also metabolized by aldose reductase to alcohol and excreted in urine. It was discussed that the CAR-linked pathway was responsible for *ca.* 15% of all metabolized ACR. Our data showed that the product 3-methylpyridinium carnosine, with concentrations of approximately 1.82 ± 0.68 nmol/mg of creatinine, is one of the most abundant acrolein metabolites in human urine. This indicates that the carnosine-linked pathway may play an important role in the cellular detoxification of aldehydes^20^.

Herein we have used MS-MSn, and NMR analysis to elucidate the structure of a 3-methylpyridinium carnosine (m/z 303) from the reaction of carnosine and acrolein. We have also developed a methodology for the simultaneous quantification of carnosine adducts in urine (Supplementary Fig. 11 is a summary of the products resulting from the reactions of carnosine with acrolein, HHE and HNE). Our results show that CAR-ACR, CAR-HHE and CAR-HNE are present in urine samples from adult humans and support the hypothesis that histidine-containing peptides are important for the metabolism of unsaturated aldehydes. A recent study has demonstrated that activation of aldehyde dehydrogenase-2 (ALDH2), which metabolizes many aldehydes, reduces nociception in two rodent models suggesting an associative role of aldehyde adducts in pain symptoms^21^. Thus the use of carnosine or the combination of carnosine with ALDH2 activators may be a promising therapeutic strategy in pain relieve or in pathologies associated with reactive aldehyde accumulation as inflammation and ischemic injury.

## Additional Information

**How to cite this article**: Bispo, V. S. *et al.* Structural Elucidation of a Carnosine-Acrolein Adduct and its Quantification in Human Urine Samples. *Sci. Rep.* **6**, 19348; doi: 10.1038/srep19348 (2016).

## Supplementary Material

Supplementary Information

## Figures and Tables

**Figure 1:**
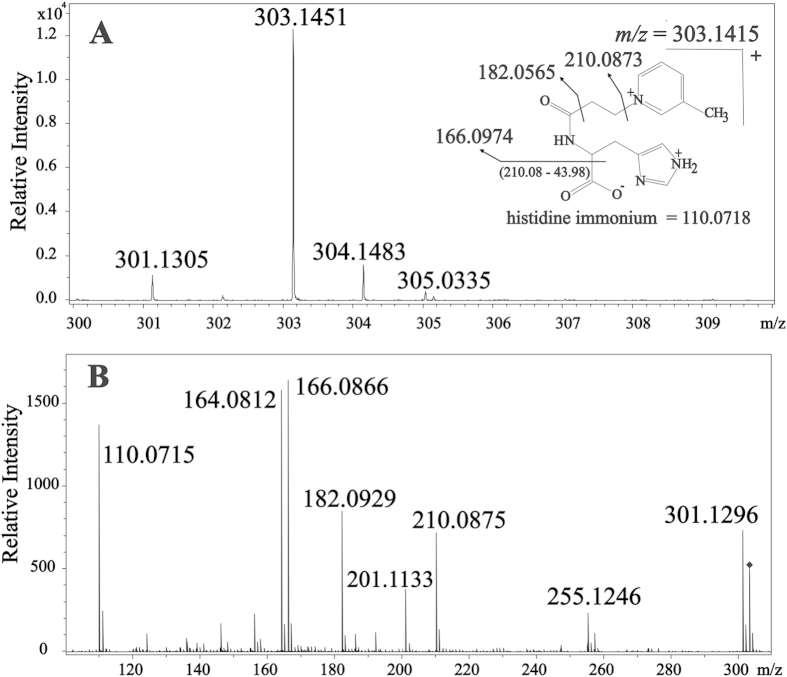
Fragmentation profile of the carnosine-acrolein adduct *m/z* = 303. (**A**) MS in the positive mode of the ion *m/z* = 303; (**B**) fragmentation profile of *m/z* = 303.

**Figure 2:**
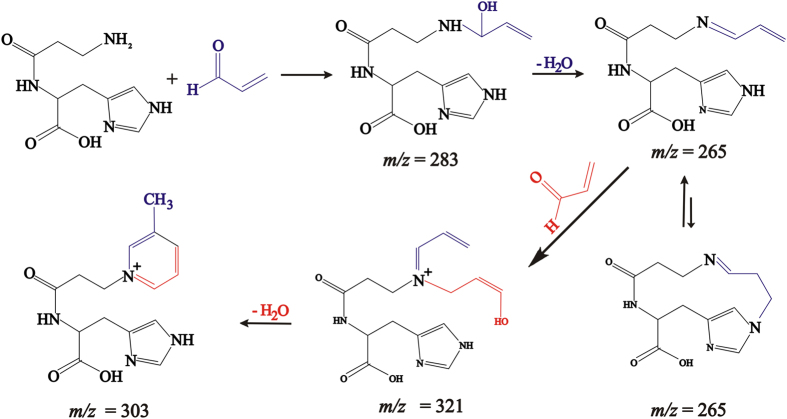
Proposed mechanistic pathway for the reaction between carnosine and acrolein.

**Figure 3:**
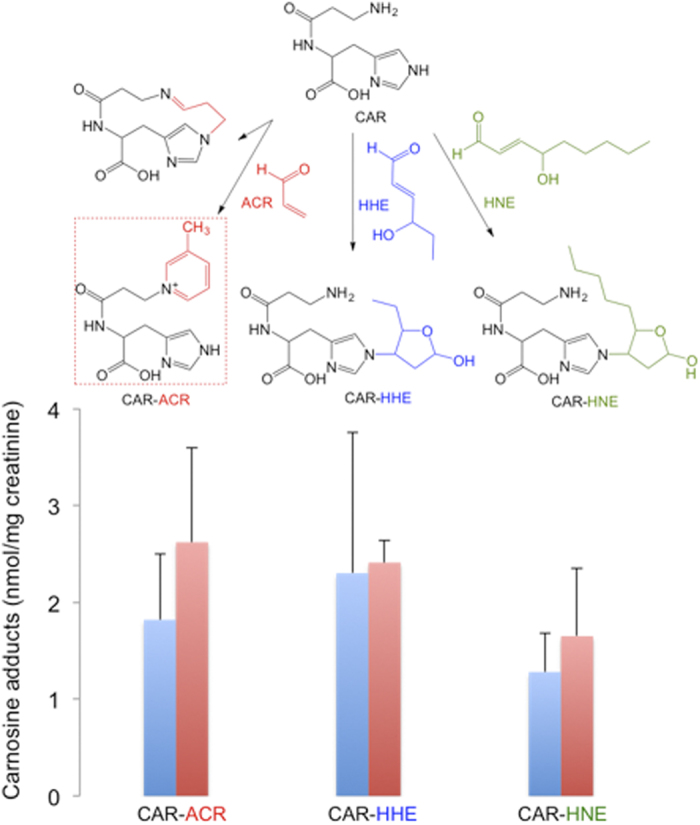
Concentrations of carnosine adducts in human urine samples. Urine (125 μL) was mixed with 125 pmol CAR-HHEd_5_, 50 pmol CAR-HNEd_11_ diluted in 50 μL of ammonium formate 5 M, and 75 μL of water (final volume 250 μL, pH 7.0). The data are expressed as the mean ± standard deviation, n = 9 men (blue bars) and 6 women (red bars).
