# Supplementary material for: Structural Elucidation of a Carnosine-Acrolein Adduct and its Quantification in Human Urine Samples
Source: Sci Rep. 2016 Jan 19;6:19348. doi: 10.1038/srep19348 (PMC4726056; doi:10.1038/srep19348)
Supplement: Supplementary Information [file srep19348-s1.pdf]

Supplementary Information for

Structural Elucidation of a Carnosine-Acrolein Adduct and its Quantification in Human Urine Samples

Vanderson S. Bispo, Ivan P. de Arruda Campos, Paolo Di Mascio and Marisa H. G. Medeiros*

1 – Departamento de Bioquímica, Instituto de Química, Universidade de São Paulo, São Paulo, São Paulo, Brazil.

2 – Instituto de Ciências Exatas e Tecnologia, Universidade Paulista, São Paulo, São Paulo, Brazil.

Corresponding author : mhgdmede@iq.usp.br

Contents

-Supplementary Text

- Ethics statement

- Urine collection

- Creatinine quantification

-Synthesis and Purification of the Carnosine-Acrolein (CAR-ACR) Adduct

-Synthesis and Purification of the Carnosine-4-Hydroxy-2-Hexenal (CAR-HHE) Adduct

-Synthesis and Purification of the Carnosine-4-Hydroxy-2-Hexenal (CAR-HHE) Adduct

-Nuclear Magnetic Resonance (NMR) Analyses Purification and Spectroscopic Characterization of the Products Formed from the Reaction of Aldehydes with Carnosine

-Supplementary Fig. 1 – HPLC-MS analysis of the reaction mixture of carnosine with

acroleine

-Supplementary Fig. 2 - Fragmentation profile of the carnosine-HHE adduct

-Supplementary Fig. 3- Fragmentation profile of the carnosine-HNE adduct

-Supplementary Fig. 4 – Determination of the molar absorption coefficients of the adducts

-Supplementary Fig 5– ^{1}H NRM data from compound *m/z* = 303 from the reaction

carnosine and acrolein

-Supplementary Fig. 6 – 18.8 T NMR spectra of CAR-ACR

-Supplementary Fig. 7 – Proposed structures for CAR-ACR $[M+H]^+$ = 303 adduct

-Supplementary Fig. 8 - Schematic representation of the HPLC-MS/MS method

-Supplementary Fig. 9 – Representative chromatograms of the urine analysis

-Supplementary Fig. 10 – Standard curves representative of the of carnosine-aldehydes quantification

-Supplementary Fig. 11– Main products from the reactions between carnosine and unsaturated

aldehydes (acrolein, HHE or HNE).

Supplementary Text

1-Materials and Methods

Materials. L-Carnosine (β-alanyl-L-histidine), acrolein, 4-hydroxy-*trans*-2-nonenal dimetylacetal and all deuterated solvents (D_2O, DMSO d_5, pyridine d_5) were acquired from Sigma-Aldrich (St. Louis, MO, USA). 4-Hydroxy-2-hexenal was purchased from Cayman Chemical Co. (Ann Arbor, MI, USA). *trans*-4-Hydroxy-2-nonenal-5,5,6,6,7,7,8,8,9,9,9-d_{11} dimethyl acetal and *trans*-4-hydroxy-2-hexenal-5,5,6,6,6-d_5 dimethyl acetal were purchased from CDN Isotopes (Quebec, Canada). All of the solvents used were of HPLC grade and were acquired from Sigma-Aldrich (St. Louis, MO, USA).

Ethics statement. The protocol for human research was approved by the human ethics committee of the Institute of Biomedical Sciences of the University of São Paulo (891/CEP_ICB-USP) as part of the project entitled “The study of redox stress biomarkers in human blood and urine”. All work was performed in accordance with the approved protocol.

Urine collection. The subjects of this study were healthy volunteers aged 16 to 41 years recruited from São Paulo University staff and students. Samples of the first morning urine were collected by volunteers and disposed into sterile 50 mL tubes. Donors were instructed to wash the hands, penis or vulva before sample collection. Urine samples were analyzed immediately for aldehyde adducts quantification and part of the samples was storage at -80°C until creatinine analysis.

Urine preparation and extraction. The freshly collected urine samples were immediately centrifuged at 11000 rpm, for 10 min under 10 °C to remove insoluble debris. Urine (125 μL)

was mixed with 125 pmol CAR-HHEd5, 50 pmol CAR-HNEd11 diluted in 50 µL of ammonium formate 5 M, and 75 µL of water (final volume 250 µL, pH 7.0). In total, 25 µL of each sample was subsequently analyzed by HPLC coupled with mass spectrometry.

Creatinine quantification. The total quantity of creatinine in urinary samples was analyzed using a Labtest kit (Lagoa Santa, MG, Brazil) in accordance with the manufacturer's instructions. Calibration curves were generated using known creatinine concentrations and utilized to calculate the quantity (mg/mL) of this compound in each examined sample.

Synthesis and Purification of the Carnosine-Acrolein (CAR-ACR) Adduct. The synthesis of the CAR-ACR adduct was performed as described by Carini *et al.*[15] with modifications. Carnosine (45 µmol) was incubated with acrolein (15 µmol) in 1 mL of 10 mM phosphate buffer at pH 7.0. The solution was mixed at 1000 rpm at 37ºC for 24 h. The adducts were analyzed using an HPLC LC 20 series system (Shimadzu, Kyoto, Japan) coupled to an Amazon Speed electrospray ion-trap mass spectrometer (Bruker Daltonics, Bremen, Germany). A Luna NH_2 analytical column (150 mm x 3.0 mm i.d., 5 µm, 100 Å, Phenomenex, Torrance, CA, USA) at 25°C and a flow rate 0.6 mL/min was used for the analysis. Purification was performed with a Luna NH_2 semi-preparative column (250 mm x 10 mm i.d., 100 Å, 10 µm of particle size Phenomenex, Torrance, CA. USA) at 25 °C and a flow rate of 5.5 mL/min. The HPLC mobile phase consisted of ammonium formate 5 mM (A) and acetonitrile (B). The adducts were analyzed and purified using a linear elution gradient: 95 to 20% B from 0-25 min, 20 to 5% B from 25-50 min, and 5 to 95 % B from 50-60 min to re-equilibrate the column. The fraction eluting at 9.2 min with m/z = 303 was collected, lyophilized and kept at -20 °C (Supplementary Fig. 1). The product was solubilized in D_2O, DMSO d_6 or pyridine d_5 and characterized by 1D ^1H, COSY, HSQC NMR and mass spectra

analyses. The exact mass was determined by injecting 5 μL of a DMSO d_6 solution containing the isolated product into a maXis 3G spectrometer (Bruker Daltonics).

Synthesis and Purification of the Carnosine-4-Hydroxy-2-Hexenal (CAR-HHE) Adduct. Carnosine (50 μmol) was incubated with HHE (25 μmol) in 1 mL of 10 mM phosphate buffer at pH 7.0. The solution was mixed at 1000 rpm at 37ºC for 24 h. The adducts were analyzed using an HPLC LC 20 series system (Shimadzu, Kyoto, Japan) coupled to an Amazon Speed electrospray ion-trap mass spectrometer (Bruker Daltonics, Bremen, Germany) with a Hydro RP analytical column (250 mm x 4.6 mm i.d., 100 Å, 4 μm particle size (Phenomenex Torrance, CA. USA) connected in sequence to a Synergi RP 80 (250 mm x 4.6 mm i.d., 100 Å, 4 μm of particle size (Phenomenex) column. The analysis was performed at 20 °C, a flow rate of 0.6 mL/min and monitoring at 220 nm. The HPLC mobile phase consisted of (A) water and (B) 0.1% formic acid in 90% acetonitrile (90 mL ACN: 10 mL formic acid 0.1%). The analysis and purification of the adducts were performed using a linear elution gradient: 5 to 95 % B from 0-25 min, 95 to 5% B from 25-32 min, and 5 % B from 50-60 min to re-equilibrate the column. The fraction with m/z = 341 eluting at 19 min was collected, lyophilized and kept at -20 °C in D_2O for further analysis. The same procedure was used for the synthesis and purification of the labeled internal standard CAR-HHEd_5.

Synthesis and Purification of the Carnosine-4-hydroxy-*trans*-2-nonenal (CAR-HNE) Adduct. The synthesis of the CAR-HNE adduct was performed as described by Aldine *et al.* [20] with modifications. A benzene solution (25 μmol) of 4-hydroxy-*trans*-2-nonenal dimethyl acetal was dried in nitrogen and hydrolyzed with 50 μL of 1 mN HCl and was mixed at 1000 rpm at 4ºC for 45 min. The resulting solution was mixed at 1000 rpm at 37 ºC for 24 h with 50 μmol carnosine in 1 mL of 100 mM phosphate buffer, pH 7.0. The adducts were analyzed

using an HPLC LC 20 series system (Shimadzu, Kyoto, Japan) coupled to an Amazon Speed electrospray ion-trap mass spectrometer (Bruker Daltonics, Bremen, Germany). The analysis was performed with a Luna C 18 (2) analytical column (250 mm x 4.6 mm i.d., 5 μm particle size, 100 Å, Phenomenex, Torrance, CA) at 25 °C and a flow rate of 1.2 mL/min. Purification was performed with a Luna C 18 (2) semi-preparative column (250 mm x 10 mm i.d., 100 Å, 10 μm particle size, Phenomenex, Torrance, CA) at 25 °C and a flow rate of 5.3 mL/min. The HPLC mobile phase consisted of (A) 0.1 % formic acid and (B) 0.1 % formic acid in acetonitrile. The analysis and purification of the adducts were conducted using a linear elution gradient: 0 to 90% B from 0-40 min, 90 to 0% B from 40-45 min, and 0 % B from 45-55 min to re-equilibrate the column. The fraction with *m/z* = 383 (9 min) was collected, lyophilized and kept at -20 °C in D_2O for further analysis. The same procedure was used to synthesize and purify the labeled internal standard CAR-HHEd_{11}.

Nuclear Magnetic Resonance (NMR) Analysis. After purification, the products isolated from the reactions of carnosine and HHE or HNE were analyzed by 1D ^{1}H NMR and COSY experiments acquired in a Bruker DRX 500 spectrometer, operating at 11.8 T. The product with *m/z = 303* obtained from the reaction between carnosine and acrolein was analyzed using 1D ^{1}H NMR, COSY and HSQC experiments performed in a Bruker AVANCE 800 spectrometer, operating at 18.8 T.

Supplementary Results

Purification and Spectroscopic Characterization of the Products Formed from the Reactions of the Aldehydes with Carnosine. Direct infusion-Ion-Trap MS and ESI-Ion-TrapMS$_n$ were used for the analysis of the products of the reactions between carnosine and CAR, HNE or HHE. The reactions with HNE and HHE gave rise to the same main products

that have been previously described.[13,16-17] The molar extinction coefficients of the products were determined by UV measurements as $\varepsilon_{260} = 2.5037 \times 10^2\ M^{-1}cm^{-1}$ (CAR-HHE) and $\varepsilon_{266} = 1.9212 \times 10^2\ M^{-1}cm^{-1}$ (CAR-HNE).

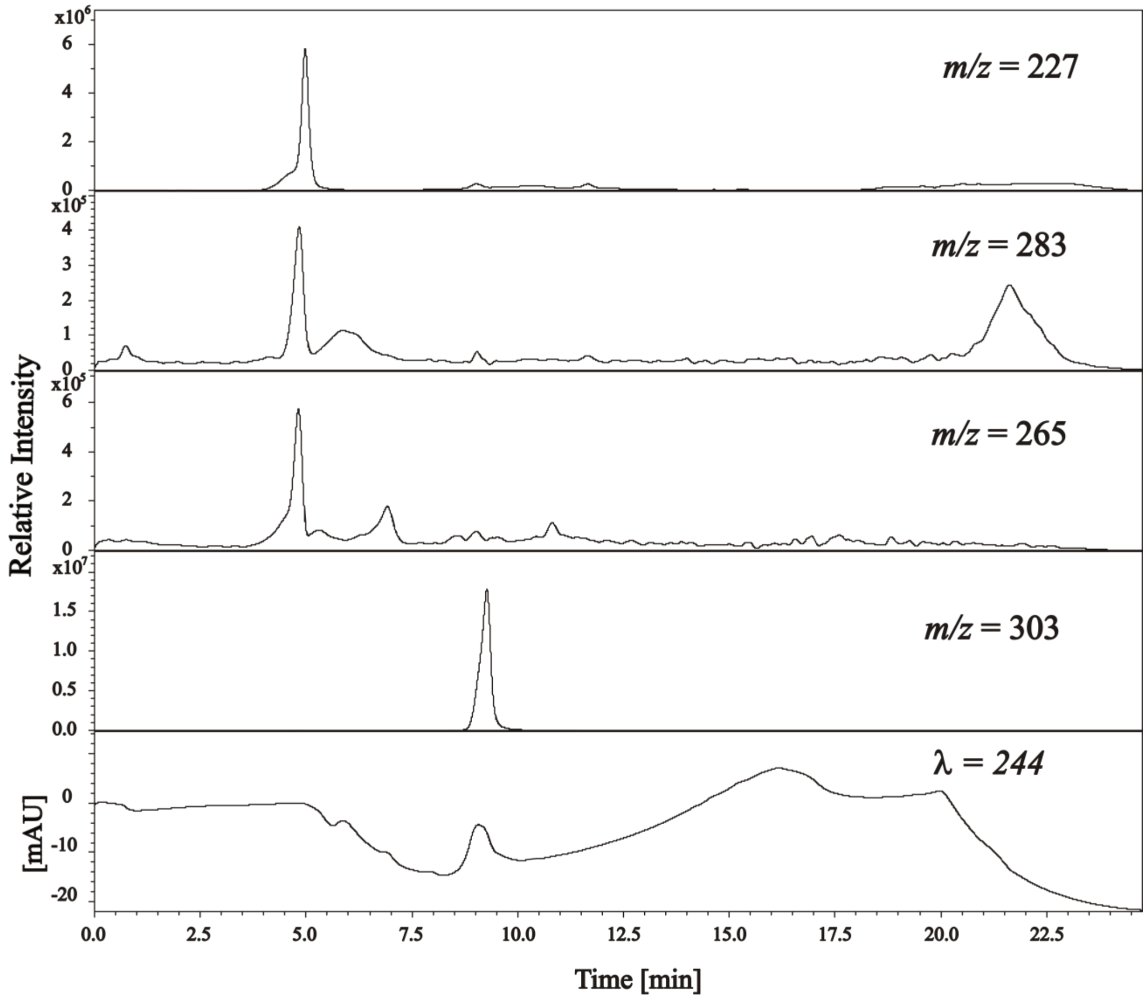

Supplementary Fig. 1 – HPLC-MS analysis of the reaction mixture of carnosine with acrolein. Reaction mixture (10 mL) was analyzed by the HPLC/MS as describes in experimental section and the peak at 9.2 min with m/z = 303 was collected.

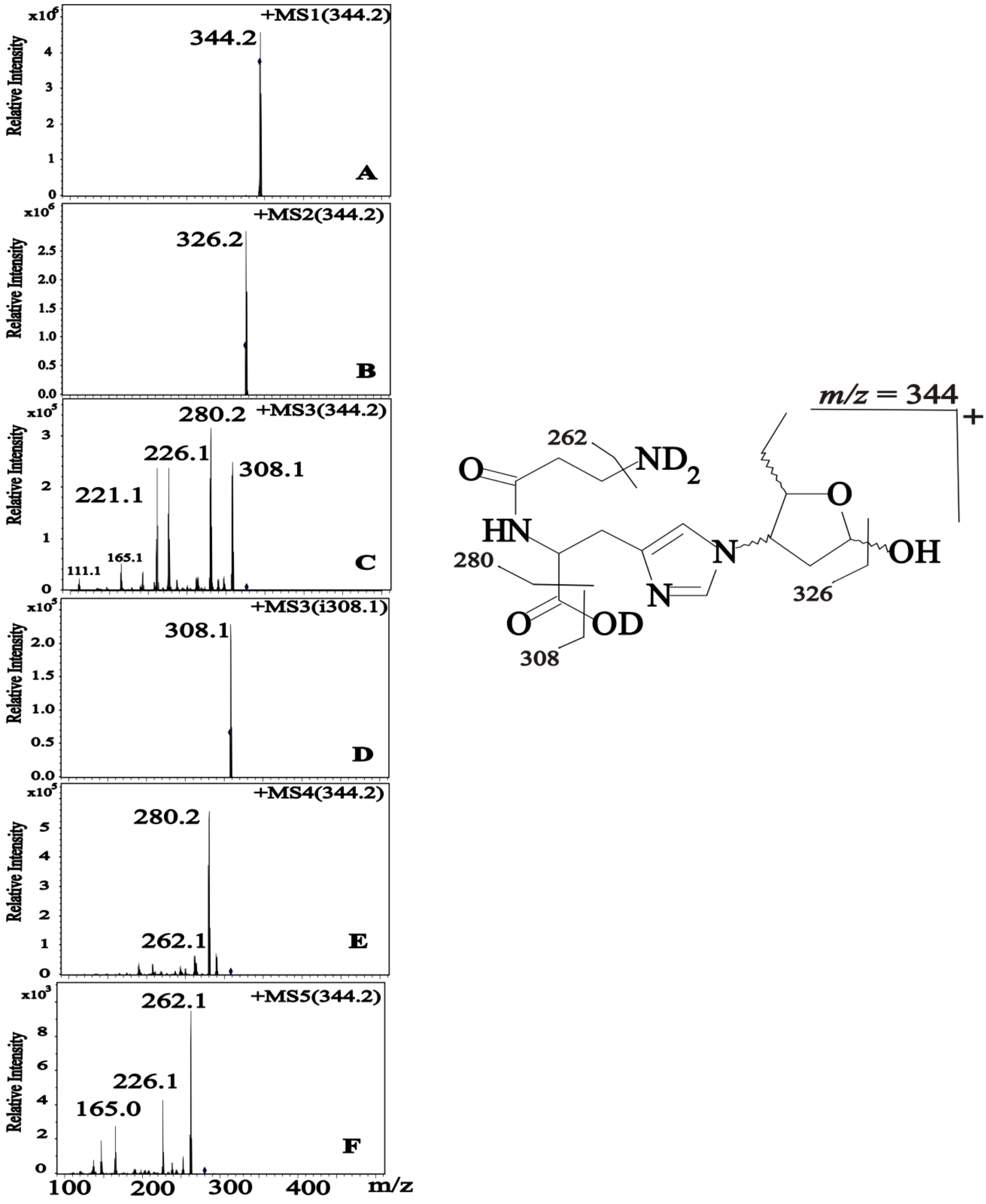

Supplementary Fig. 2 - Fragmentation profile of the carnosine-HHE adduct. A – ion m/*z* = 344 (isolated); **B** – fragmentation of the *m/z* = 344; **C** – fragmentation of the *m/z* = 326.2; **D** – ion m/*z* = 308 (isolated); **E** – fragmentation of the *m/z* = 308.1; **F** – fragmentation of the *m/z* = 280.2. The adduct was dissolved and stored in D_2O. The results were obtained in an Amazon Speed ion trap from Bruker Daltonics with direct infusion (5 mL/min) of the isolated compounds in solution (100 pmol/ mL) and the electrospray source in positive mode (ESP^+), 4500 V.

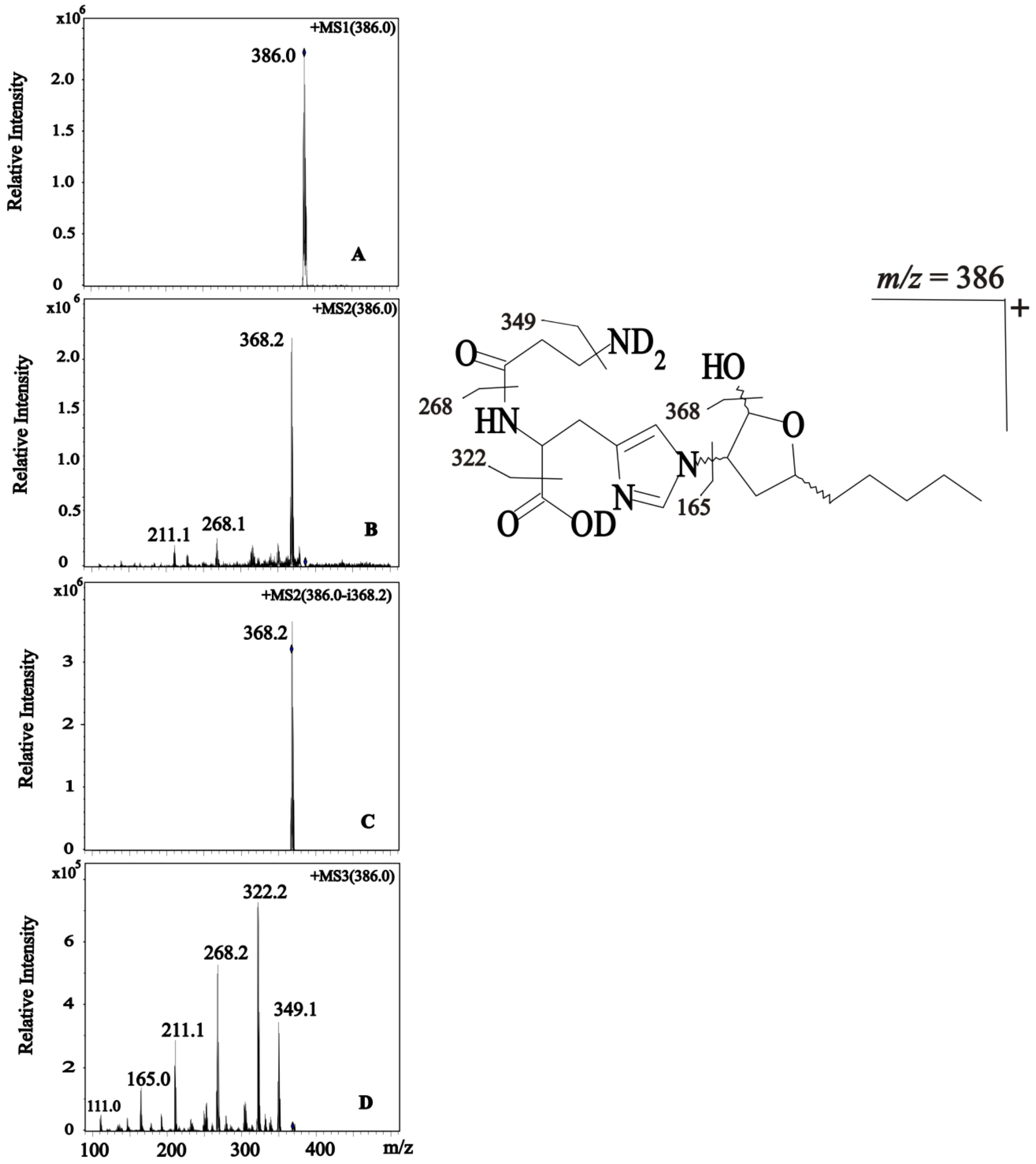

Supplementary Fig. 3 – Fragmentation profile of the carnosine-HNE adduct. A – ion m/*z* = 386 (isolated); **B** – fragmentation of the *m/z* = 386; **C**– ion m/*z* = 368.2 (isolated); **D** – ion m/*z* = 308 (isolated). The adduct was dissolved and stored in D_2O. The results were obtained in an Amazon Speed ion trap from Bruker Daltonics with direct infusion (5 mL/min) of the isolated compounds in solution (100 pmol/ mL) and the electrospray source in positive mode (ESP^+), 4500 V.

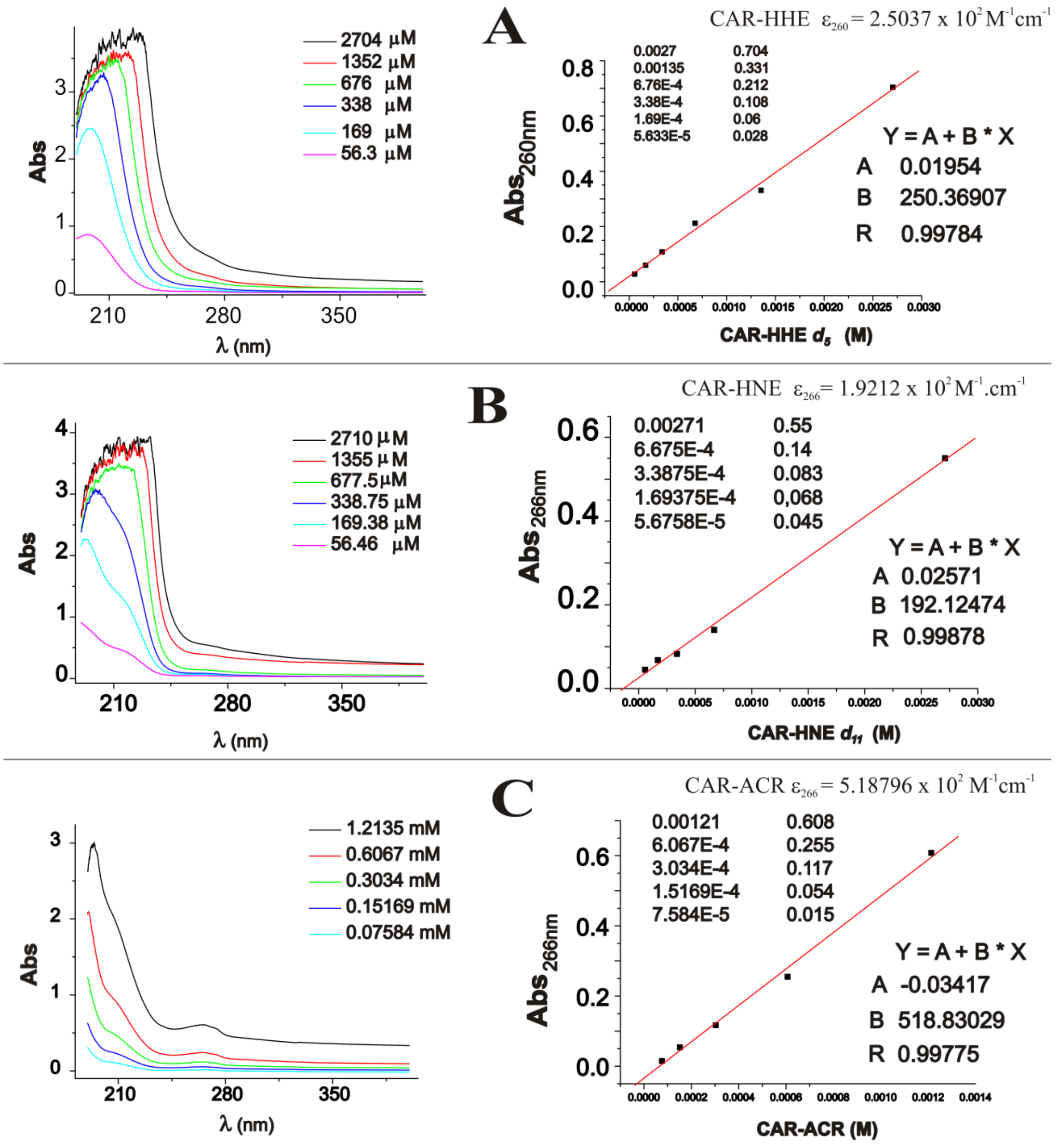

Supplementary Fig. 4 – Determination of the molar absorption coefficients of the adducts formed from the reaction of carnosine with unsaturated aldehydes. A – CAR-HHE, **B** –CAR-HNE, **C** – CAR-ACR 303. Solutions with different concentration of each purified adduct were prepared and their absorption spectra between 400 – 200 nm were recorded. The molar absorption coefficients (e) were determined from linear regression analysis of the absorbance and adduct concentration data.

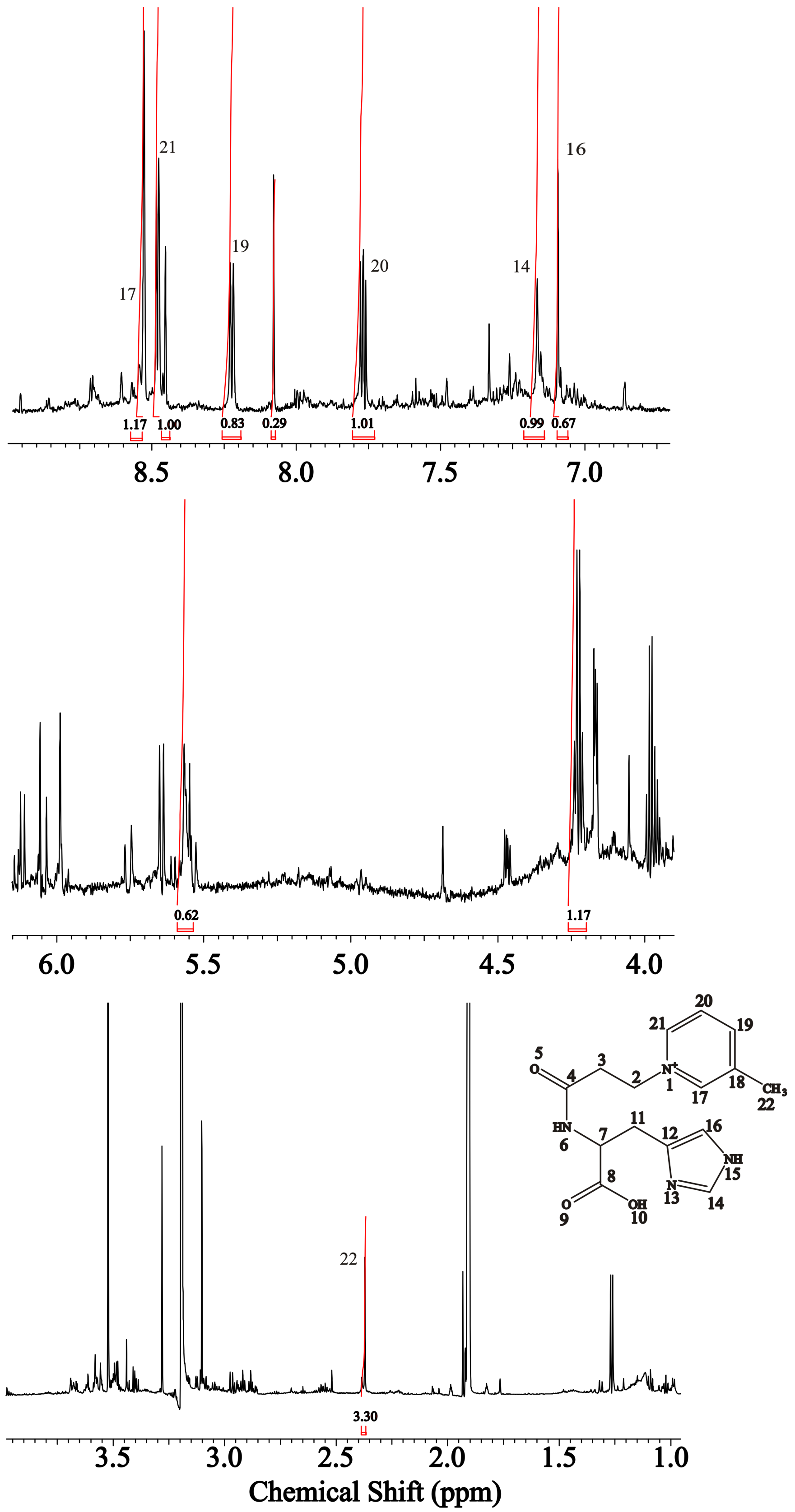

Supplementary Fig. 5 – [1]H NRM data of compound m/z = 303 from the reaction carnosine and acrolein. 1.5 mg of the compound CAR-ACR was synthesized, dissolved in 600 mL D_2O + 30 mL de acetic acid *d_4* and analyzed on a Bruker Avance 800 instrument.

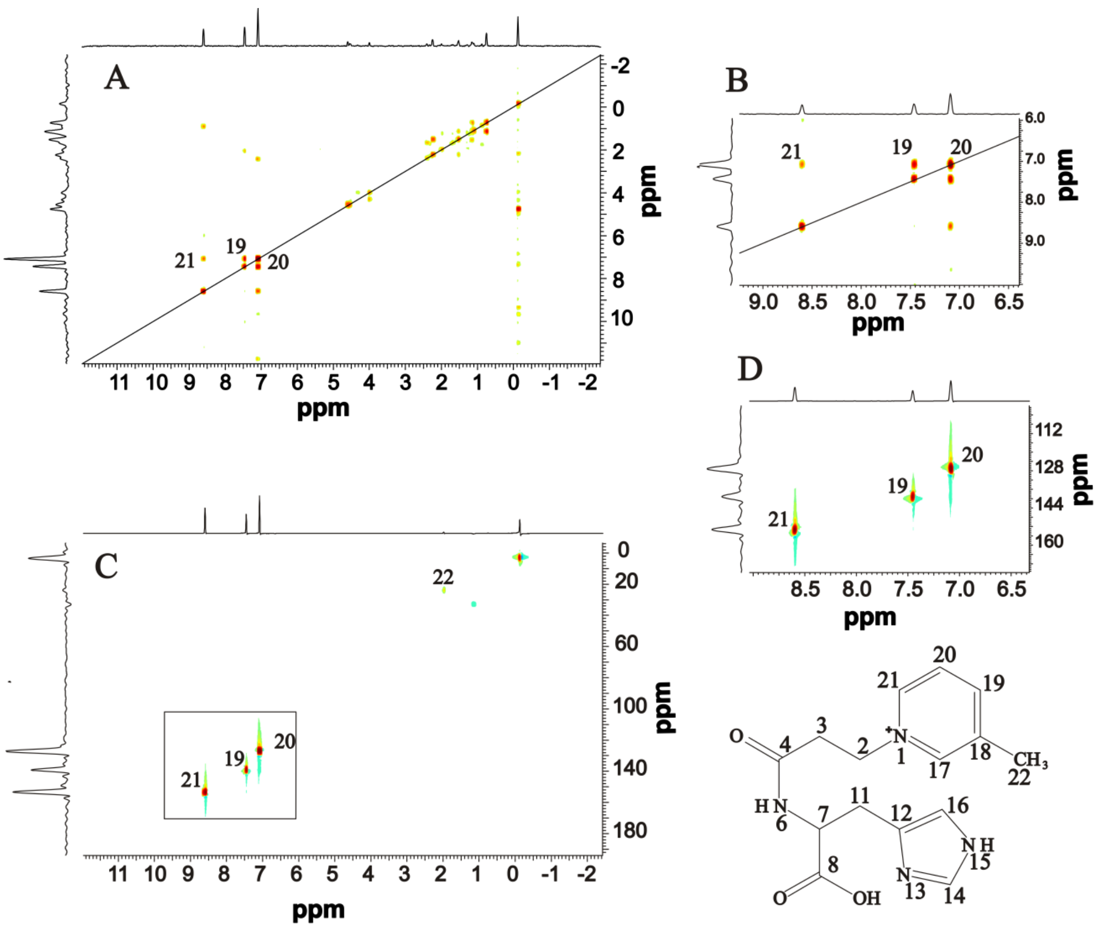

Supplementary Fig. 6 – 18.8 T NMR spectra of CAR-ACR *m/z* = 303. A and B – MNR ^{1}H COSY; **C and D** HSQC. CAR-ACR m/z 303 (1.0 mg) dissolved in 600 mL of pyridine-d_5 with 20 mL of acetic acid-d_4 and analyzed on a Bruker AVANCE 800 spectrometer.

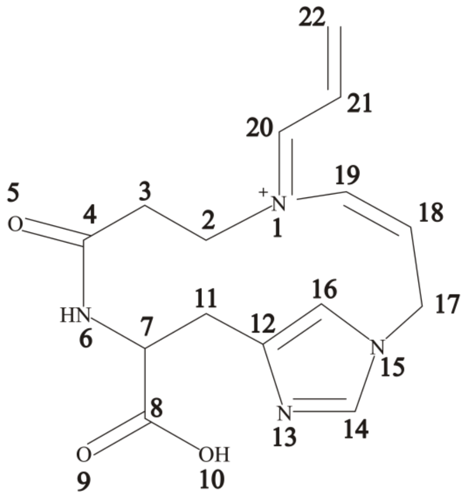

Carine et al., 2003

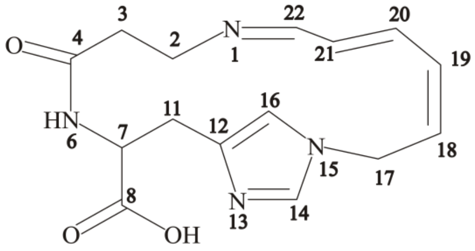

(I)

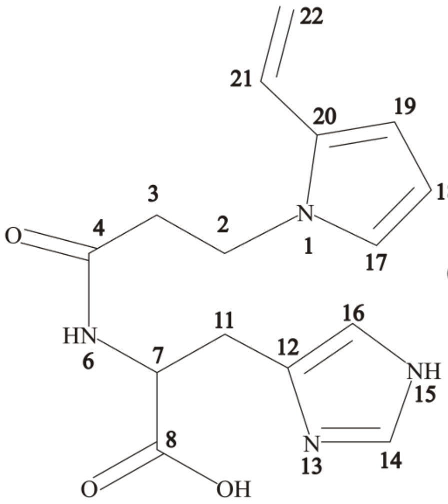

(II)

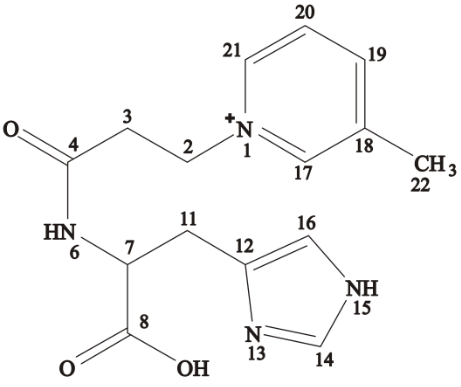

(III)

Supplementary Fig. 7 – Proposed structures for CAR-ACR $[M+H]^+$ = 303 adduct. These structures have molecular formula $C_{15}H_{19}N_4O_3$, *m/z* = 303.1451 (as calculated by Compass IsotopePattern 3.2 software, Bruker Daltonics, Bremen, Germany) and were proposed in the literature as the product from CAR-ACR reaction.[14-16] The structure suggested before[15] does not fit in the fragmentation profile and the ^{1}H NMR data obtained in our studies. Structure I have one hydrogen bound to nitrogen (^{1}H NMR data) and the coupling of the carbon 18 – 21 does not match our NMR data, whereas structure III does.

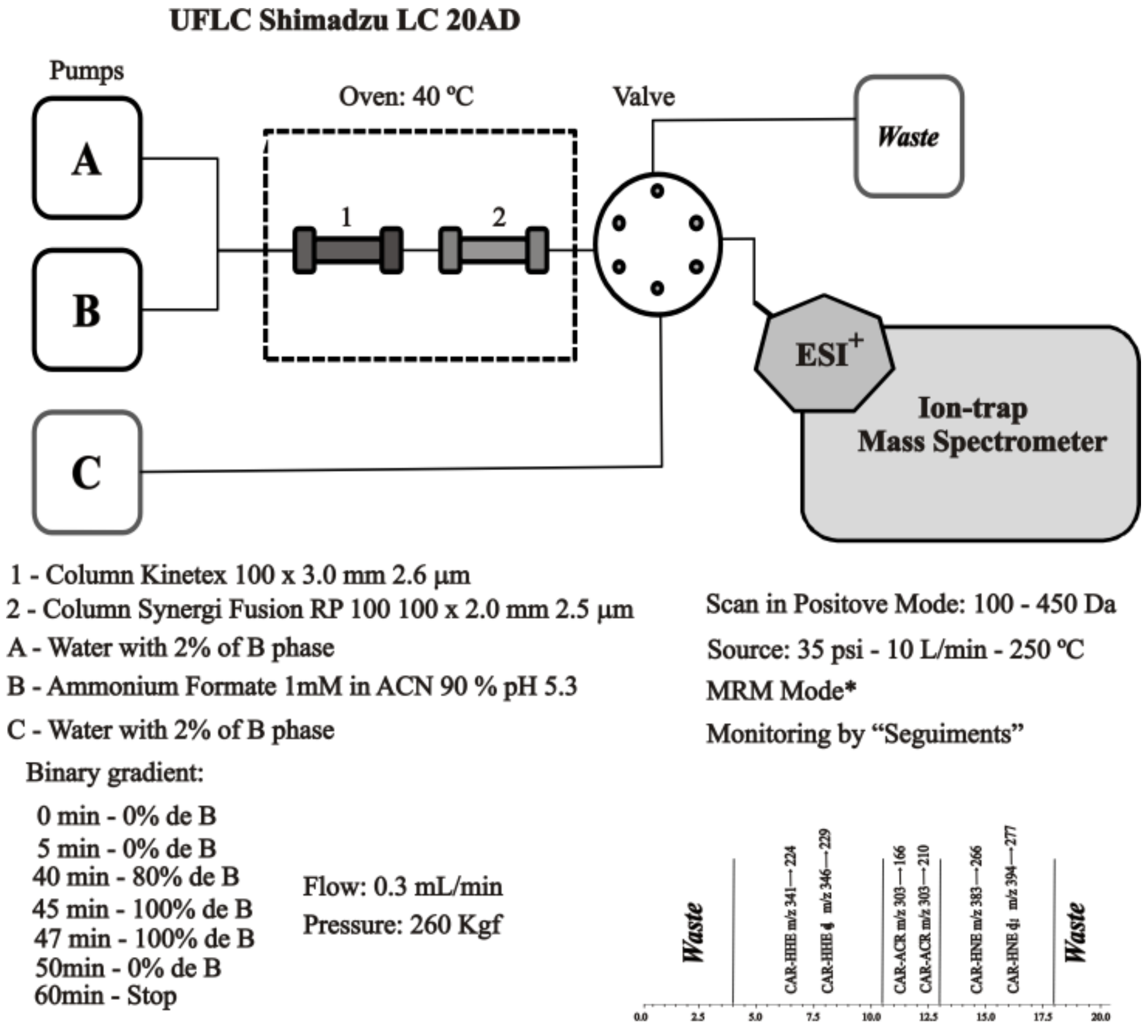

Supplementary Fig. 8 - Schematic representation of the HPLC/MS/MS method.

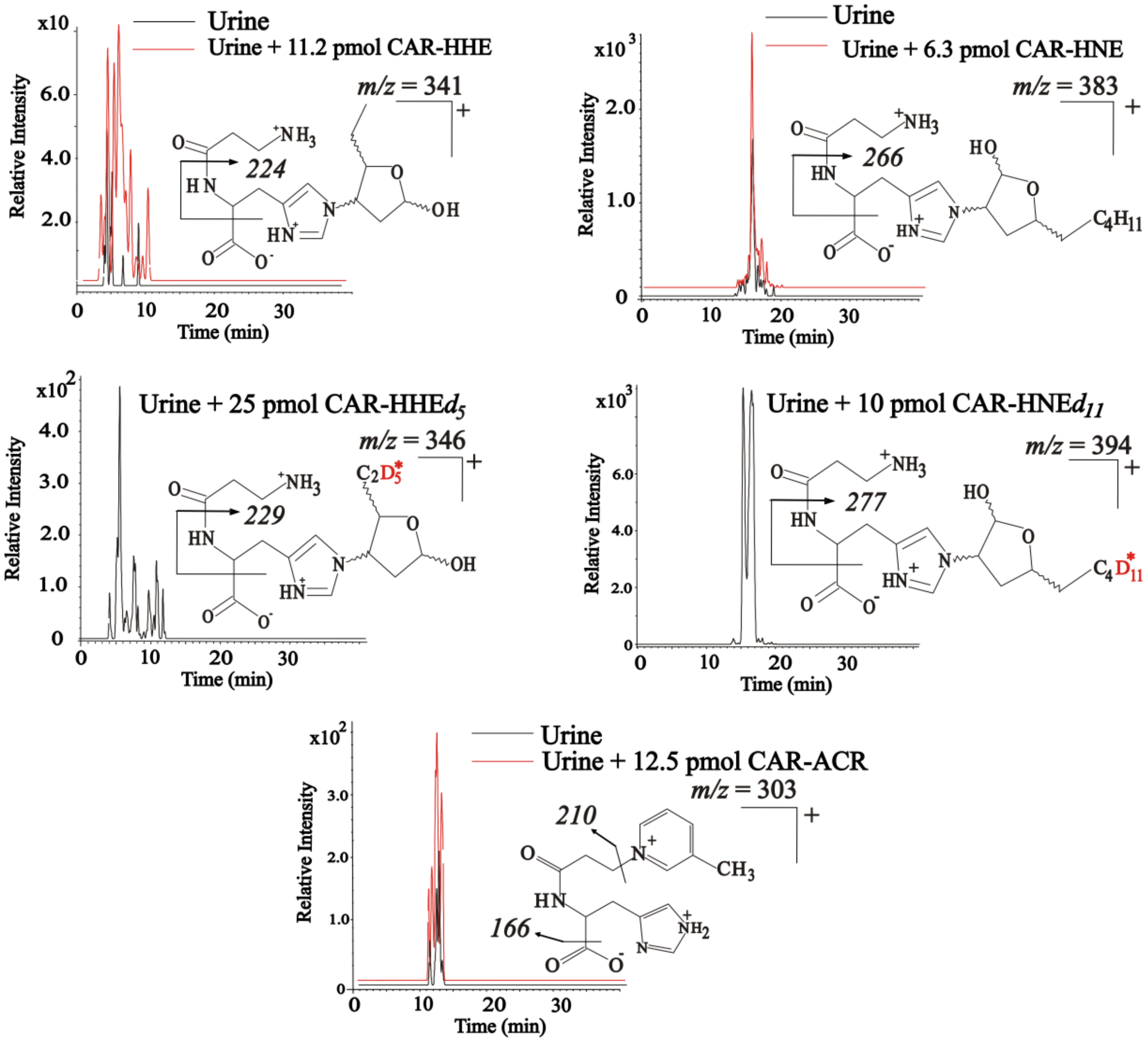

Supplementary Fig. 9 – Representative chromatograms of the urine analysis. Urine (125 mL) with 50 mL of ammonium formate 5 M and 75 mL of water (final volume 250 mL, pH 7.4) (black line). Urine (125 mL) with 50 mL of ammonium formate 5 M and CAR-HHEd_5; CAR-HNEd_{11}, or CAR-ACR adducts (red line). The conditions were as described in Supplementary Fig. 8.

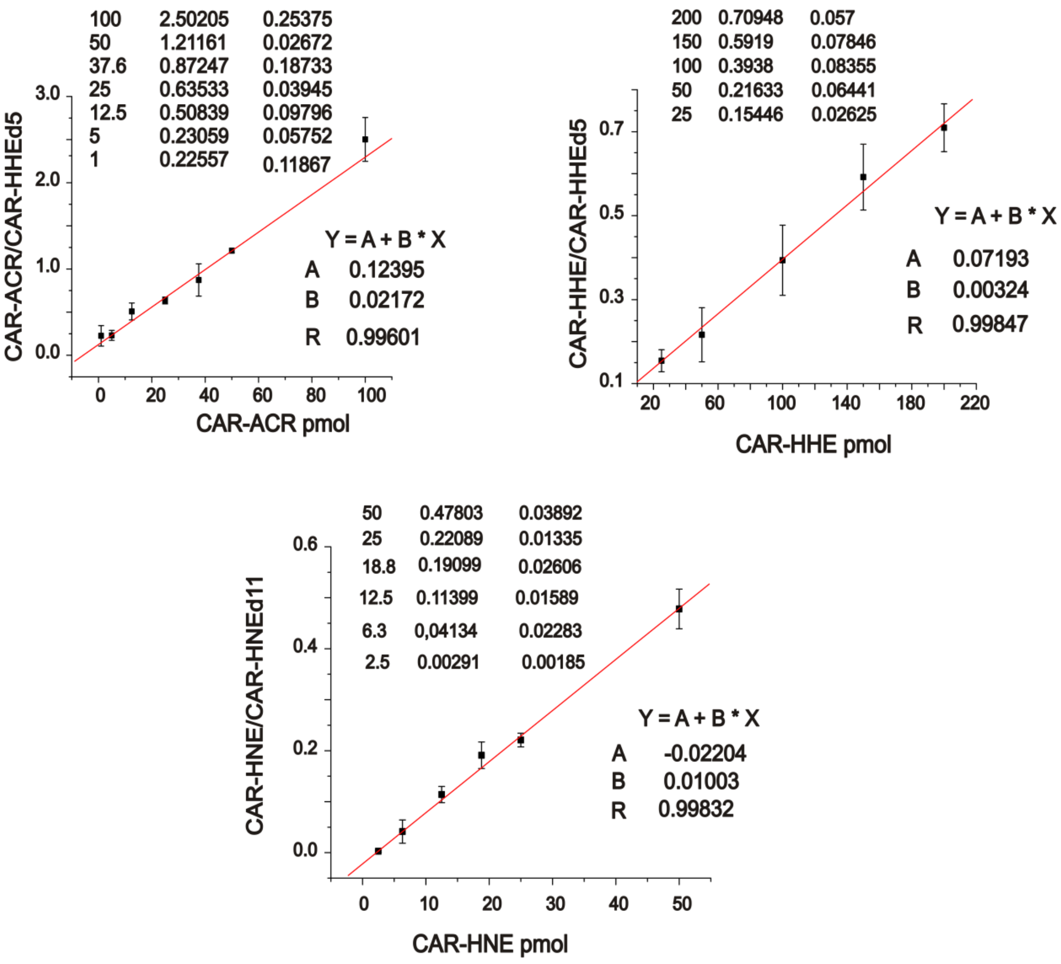

Supplementary Fig. 10 – Standard curves representative of the of carnosine-aldehydes quantification. Human urine (125 mL) was mixed with 125 pmol CAR-HHEd$_5$, 50 pmol CAR-HNEd$_{11}$, 50 mL ammonium formate 5 M and 75 mL of a carnosine-adducts in different concentrations (final volume 250 mL, pH 7.4). Solutions (25 mL) were injected in system an HPLC LC 20 series (Shimadzu, Kyoto, Japan) coupled with an Amazon Speed electrospray ion-trap mass spectrometer (Bruker Daltonics, Bremen, Germany) in the positive mode (ESI$^+$), 4500 V on capillary, 35 psi of nebulizer gas, 10 L/min of dry gas and 250 ºC dry temperature, in a with a system with two columns in sequence: first a Kinetex C18 (100 mm x 3 mm i.d., 2.6 µm of particula size, 100 Å, Phenomenex, Torrance, CA) followed by a Synergy Fusion RP Polar (100 mm x 2 mm i.d., 2.5 µm of particula size, 100 Å, Phenomenex, Torrance, CA), under 40 °C and flow 0.3 mL/min. As mobile phase was used (A) water with 2 % of B phase (v/v) and (B) ACN 90% with ammonium formate 1mM pH 5.3. CAR-HHEd$_5$ and CAR-HNEd$_{11}$ were used as internal standard (IS). The standard curves and the quantification in biological samples were obtained by the relation *area compound /area Internal Standard*. The coefficient of variance (CV) was calculated by the relation *standard deviation /average* and the accuracy by software DataAnalisis 4.1 ((Bruker Daltonics, Bremen, Germany).

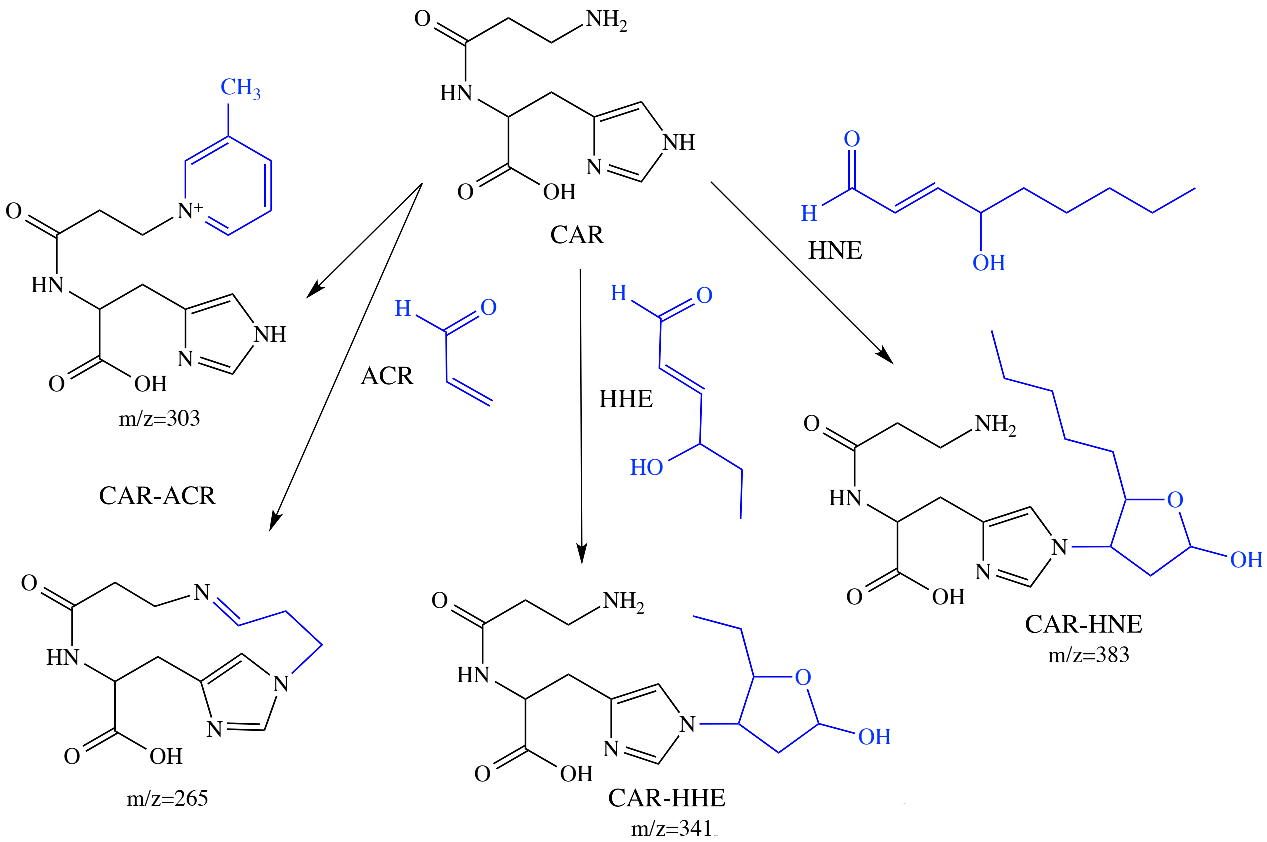

Supplementary Fig. 11– Main products from the reactions between carnosine and unsaturated aldehydes (acrolein, HHE or HNE).
